# Attentional disengagement, craving, and mentalizing: a preliminary experimental study among older-aged male gamblers

**DOI:** 10.1186/s40359-024-01651-7

**Published:** 2024-03-19

**Authors:** Maria Ciccarelli, Barbara Pizzini, Mark D. Griffiths, Marina Cosenza, Giovanna Nigro, Francesca D’Olimpio

**Affiliations:** 1 Department of Psychology, Università degli studi della Campania “Luigi Vanvitelli”, Viale Ellittico, 31, Caserta, 81100 Italy; 2 Università Giustino Fortunato, Via Raffaele Delcogliano, 12, Benevento, 82100 Italy; 3 Psychology Department, Nottingham Trent University, 50 Shakespeare Street, Nottingham, NG1 4FQ UK

**Keywords:** Gambling, Older-aged gambling, Attentional bias, Disengagement bias, Craving, Mentalizing

## Abstract

**Introduction:**

Empirical studies have demonstrated the role that attentional bias, the mutual excitatory relationship between attentional bias and craving, and mentalizing play in problem gambling. Although problem gambling rates among older-aged adults have steadily increased in recent years, research studies among this cohort are scarce. The present study is the first to empirically investigate attentional bias, as well as the joint role of attentional bias, craving, and mentalizing among older-aged gamblers.

**Method:**

Thirty-six male older-aged gamblers were administered the South Oaks Gambling Screen (SOGS), the Gambling Craving Scale (GACS), and the Reflective Functioning Questionnaire (RFQ-8) to assess gambling severity, craving levels, and mentalizing, respectively. Participants also performed a modified Posner Task to investigate attentional biases.

**Results:**

Hierarchical linear regression analysis showed that among older-aged male gamblers, GACS Anticipation and RFQ-8 Uncertainty about mental states, as well as disengagement bias at 100 ms, significantly predicted gambling severity.

**Conclusion:**

The present study provides the first empirical support for the role of attentional bias, craving, and mentalizing among older-aged gambling. More specifically, a difficult in disengaging attention away from gambling, the anticipation of pleasure deriving from gambling, and hypomentalizing predicted gambling severity among older-aged gamblers. The findings make an important contribution, by identifying the factors responsible for problem gambling among this specific age cohort and suggesting that timely interventions for mentalizing and attentional bias may be necessary to prevent problem gambling in old age.

## Introduction

Problem gambling rates among older-aged adults have steadily increased in recent years [1]. However, to date, there is a dearth of experimental research on this topic (for a review, see [2]). According to a recent review, the paucity of studies on gambling behaviors in old age is ascribable to different sources of bias that have produced an apparent low prevalence of problem gambling among older-aged adults (compared to younger cohorts), resulting in an underrepresentation of older adults in gambling research [3]. These biases include social desirability related to the stigmatization of gambling behaviors among older-aged adults, the deficits in recalling gambling experiences and/or potential symptoms of gambling disorder, and selection bias including the increased use of online surveys that are likely to exclude older people from studies.

Several theoretical models posit that attentional bias plays a central role in gambling disorder [4, 5]. Attentional bias comprises enhanced attentional processing of addiction-related (compared to matched neutral) stimuli [6], which can result in (i) facilitated detection of addiction stimuli, (ii) a difficult disengagement from the addiction stimuli, and/or (iii) an avoidance of addiction stimuli. In the addictions field (and more specifically, the gambling addiction field), the important role of the mutual excitatory relationship between attentional bias and craving has been posited, which relates to the strong subjective desire to gamble [6–13]. The repeated association of gambling stimuli with reward can make gambling stimuli salient through the classical conditioning process. Consequently, this encourages all gambling activity-seeking behaviors and can contribute to the risk of gambling addiction. The mechanism through which salient stimuli captures the individual’s attention, increases craving, and causes them to gamble should be considered in light of the fact that attention is a limited resource and that the salience of gambling stimuli, combined with craving, causes the individual’s interests to be completely absorbed by gambling.

Previous empirical research has focused upon the identification of the attentional mechanisms responsible for gambling across different degrees of gambling involvement severity [5, 14–16]. Although there are a few exceptions, the findings have consistently indicated that individuals with gambling problems preferentially attend to gambling cues over competing stimuli not related to gambling [17–20]. The first studies on the topic, using the Stroop task, observed that individuals with gambling problems respond more slowly to gambling-related words or images, as compared to controls [21–23], especially when the gambling stimuli were related to their favorite type of gambling activity [15, 22]. Given that Stroop tasks capture a general bias for gambling stimuli, without differentiating between orienting and maintenance of attention [24], subsequent studies used other tasks such as visual dot probe tasks.

Using these tasks, Vizcaino et al. [25] found that individuals with gambling problems reacted quickly to dots following gambling than non-gambling images at exposure times that are indicative of maintenance of attention. Another attempt to overcome the limitations of the Stroop was made by Brevers et al. [26] with the flicker paradigm, where participants were required to identify the difference (gambling or non-gambling cues) between a couple of pictures. The authors observed that individuals with gambling problems were faster in orienting to (but slower to disengage from) gambling stimuli, as compared to the control group. Using a rapid serial visual presentation paradigm to investigate the spatial attentional for gambling stimuli among high-risk and low-risk gamblers, Hudson et al. [14] found that high-risk gamblers showed a preference for gambling stimuli at the level of maintenance of attention, relative to distractors such as emotional and neutral stimuli. Other studies that have recorded participants’ eye movements through a direct measure of attentional bias, such as the eye tracking technology, have observed that individuals with gambling problems fixate on gambling images earlier than other images [15, 27], and preferentially attend to them for a significant longer period of time compared to controls [15, 16, 28].

A series of study conducted by Ciccarelli and colleagues [29–32] used a modified version of the Posner task. This allowed them to measure, through different exposure time of gambling and non-gambling stimuli, both initial orienting and maintenance of attention. They observed an (i) attentional facilitation in the initial engagement of attention among adults with gambling problems, (ii) attentional facilitation in the maintenance of attention among adolescents with gambling problems, and (iii) avoidance bias in the maintenance of attention among gamblers in treatment. They hypothesized that attentional bias could reflect the degree of gambling severity and involvement. More specifically, while the automatic orientation of attention towards gambling stimuli might be a consequence of repeated exposure to such stimuli, and one of the factors responsible for continuing gambling, the intentional distraction from gambling stimuli could be a strategic attempt to remain abstinent [30].

The heterogeneity of the attentional paradigms employed does not always allow for a direct comparability of the results obtained. However, studies in the extant literature consistently demonstrate that the spatial attention of individuals with gambling problems is preferentially directed towards gambling cues in the initial orienting [26, 29, 30] and/or in the maintenance of attention [15, 25, 26, 28, 33]. Despite this consistency in findings, it should be noted that the aforementioned studies only recruited young adults ([15, 16]; see [5] for a review) or adolescents [31], substantially neglecting the role of attentional bias in older-aged gamblers.

Moreover, among the studies that have examined the relationship between attentional bias and craving, some observed that high levels of craving are associated with high attentional bias scores, suggesting that the desire to gamble could enhance the detection of gambling stimuli [29, 30, 33], whereas others did not find support for this association [26, 31, 33]. However, no previous studies have explored this association among older-aged gamblers.

Importantly, and to the best of the present authors’ knowledge, there are no prior studies that have examined mentalizing and gambling among older-aged gamblers. The construct of mentalization has gained increasing research attention. According to Bateman and Fonagy [34], mentalization can be defined as the mental process whereby individuals attribute meaning to their own and others' behavior by questioning (not necessarily explicitly and consciously) the mental states that motivates behaviors, such as feelings, beliefs, needs, etc. Examining the relationship between mentalizing and gambling among those who are older-aged could be of particular importance in relation to studies reporting a positive association between impaired mentalizing abilities and gambling among adults and adolescents [35–40], as well as studies that, evaluating the changes in mentalizing skills across adult lifespan, have reported an age-related decline of mentalizing abilities from the fifth decade of life onwards [41, 42]. From this perspective, compromised mentalizing might constitute an additional risk factor for gambling among older-aged adults.

Given these literature gaps, the present study is the first to empirically investigate attentional bias among older-aged gamblers, in order to identify which attentional component and what type of bias contributes to problem gambling among older-aged gamblers. Because attentional biases are deeply connected to the experience of craving, and craving has not been previously investigated among older-aged gamblers, the present study also evaluated the unexplored relationship between attentional bias and craving among older-aged gamblers. Moreover, there is also no evidence concerning the role of mentalizing, as well as the joint role of attentional bias, craving, and mentalizing among older-aged gamblers. Therefore, the present study aimed to fill these gaps by investigating the interplay between these factors.

Based on previous attentional bias research, facilitated attention for gambling stimuli, craving, and hypomentalizing were expected to predict gambling severity among older-aged gamblers.

## Method

### Participants and procedure

Thirty-six male gamblers,^1^  aged between 60 and 80 years (*M*_*age*_ = 67.08 years; *SD* = 5.79), recruited from several Italian gambling venues, took part in the study. Prior to participation, individuals were assured about the possibility to withdraw from the study whenever they wanted. In a quiet room of the gambling venues, participants individually and anonymously performed the modified version of the Posner Task to assess attentional bias [44], and completed the South Oaks Gambling Screen (SOGS; [45, 46]), the Gambling Craving Scale (GACS; [29, 47]), and the Reflective Functioning Questionnaire (RFQ-8; [48, 49]) to assess the degree of problem gambling severity, gambling-related craving, and mentalization, respectively. After having signed the informed consent, half of the participants completed the computerized task at the beginning of the session, and the other half at the end. In this way, the (potential) influence of the experimental task on the paper-and-pencil measures, and vice versa, was balanced. The psychometric scales were administered in counterbalanced order. After data collection, participants were debriefed about the real aims of the study and all their questions were answered. Participation in the study was voluntary and participants did not receive any reward. The ethics committee of the research team's university department approved the present study. The completion of the instruments and participation in the computerized task took approximately 25/30 min. For each measure, participants received detailed written instructions.

### Measures

The SOGS^2^ is a self-report scale that assesses gambling severity and comprises two sections. The first section includes non-scored items providing information about the frequency of participation in gambling and the largest amount of money gambled on any one day, as well as the preferred gambling activities (e.g., cards, horses, bingo, etc.), venues (e.g., bar, tobacco shop, etc.), company (e.g., friends, partners, etc.), and mode (e.g., online, offline). The second section includes 20 scored dichotomous (*yes/no*) questions, based on the DSM criteria for pathological gambling [50] and assesses the severity of gambling involvement through questions investigating chasing behavior frequency, the guilt related to gambling involvement, loss of control over gambling, etc. The maximum score is 20, with higher scores reflecting more severe gambling. More specifically, scores from 0 to 2 indicate no gambling problems, scores of 3 and 4 indicate problem gambling, and a score of 5 or above denotes (probable) pathological gambling. In the present study, the total score was used.

The Gambling Craving Scale (GACS) is a self-report scale comprising nine items that assesses, on a seven-point Likert scale (from *strongly disagree* to *strongly agree*), three different dimensions of gambling-related craving: Desire, Anticipation, and Relief. Desire refers to the immediate desire to gamble, Anticipation refers to the expectation of the positive experiences that would result from gambling, and Relief refers to the expectation of the immediate relief from negative emotional states that would result from gambling participation. Higher scores reflect stronger feelings of craving.

The eight-item Reflective Functioning Questionnaire (RFQ-8) is a self-report measure that assesses two different dimensions of mentalization: *Certainty about mental states* and *Uncertainty about mental states* on a seven-point Likert scale (from *strongly disagree* to *strongly agree*). Low scores on Certainty scale reflect inaccurate mentalizing (i.e., hypermentalizing) while high scores indicate genuine mentalizing. Low scores on Uncertainty scale reflect genuine mentalizing, while high scores indicate a lack of knowledge about mental states (i.e., hypomentalizing).

The modified version of the Posner Task is a computerized task detecting attentional bias, administered on a PC using the experimental software SuperLab 4.0 and the operating system Windows 8. Color pictures (*N* = 40), chosen from non-copyrighted images on the internet, were used as stimuli (i.e., 20 gambling-related pictures and 20 neutral pictures). The gambling and neutral pictures were matched for both color and shape (for example, a slot machine was matched with petrol pump, gambling chips with buttons, and a roulette wheel with a wall clock). Each picture measured 350 × 350 pixels and was displayed on a grey background of a personal computer with a 15.6″ monitor. The task consisted of 160 trials, 80% of which (i.e., 128 trials, 64 gambling and 64 neutral) were valid, while the remaining 20% (32 trials,16 gambling and 16 neutral) were invalid. In the valid trials, the target (dot) appeared in the same location of the image that preceded it, while in the invalid trials the target was presented on the opposite side [44].

Each trial started with a fixation point (“ + ”) (ITI;1 cm in height) presented for 1000 ms, followed by a picture, in the left or right side of the screen for a fixed period of 100 or 500 ms, after which it was immediately substituted by a dot (target). The dot was blue and appeared in the same position of the picture (valid trial) or on the opposite side (invalid trial) for 1500 ms. Each image was displayed four times, as a valid and invalid trial, for 100 ms and 500 ms. The manipulation of the duration of the stimulus allows the assessment of two attentional components: the initial orienting of attention (from 50 to 200 ms), and maintenance of attention (from 500 ms and upwards) [6, 51, 52]. Participants, seated 60 cm from the monitor and level with the center of the screen, were requested to press a button on a keyboard (marked with white stickers) based on the location of the appearance of the probe: “a” for left and “ù” for right. Participants were tested individually and instructed to respond to the probe as quickly and accurately as possible. Both accuracy and response times (RTs) were recorded.

### Data preparation

Based on reaction times (RTs) for correct responses, facilitation bias, disengagement bias, and avoidance bias were calculated. Facilitation bias was calculated by subtracting RTs for gambling-related stimuli from neutral stimuli in valid trials. Positive scores indicate a facilitated detection of gambling rather than neutral images. Disengagement bias was calculated by subtracting RTs for neutral stimuli from gambling-related stimuli in invalid trials. Positive scores indicate a prolonged attention on gambling rather than neutral images. Negative scores of both facilitation and disengagement bias indicate an avoidance (i.e., a tendency to divert attention from gambling stimuli). Values close to zero indicate a lack of attentional biases (i.e., no differences between the attentional processing of gambling and neutral pictures).

### Statistical analysis

Data were analyzed with the IBM Statistical Package for the Social Sciences, version 20.0. The alpha significance level was set at 0.05. All variables were initially screened for missing data, distribution abnormalities, and outliers [53]. To examine the relationships between the study variables, bivariate correlations were performed. To identify the predictors of problem gambling among older-aged gamblers, a hierarchical linear regression analysis was performed with the SOGS total score as the dependent measure, using age (Step 1), GACS subscales scores (Step 2), RFQ-8 subscales scores (Step 3) and attentional bias (facilitation and disengagement at both 100 ms and 500 ms; Step 4) as predictors. To control for the presence of multicollinearity, the variance inflation factors (VIF) was calculated for each predictor and, if present, the predictor was excluded from subsequent analysis. In the present study, the VIF was below the recommended cut-off of 2.5 [54], indicating no issues with multicollinearity.

## Results

Almost nine-tenths of the participants (88.39%) were regular gamblers (i.e., gambled at least once a week). More specifically, one-third of the sample regularly gambled both online and offline (38.39%), 36.11% regularly gambled mainly offline, and 13.89% regularly gambled mainly online. The majority of the sample reported a gambling onset before 30 years (81.3%; Mean = 25.03; SD = 11.20). The most popular gambling venues were tobacco shops (44.4%), home (41.7%), bars (38.9%), and betting centers (36.1%). Participants preferred gambling alone (52.8%) or with friends (47.2%). The most reported motivations for gambling (participants could report more than one motivation) were: entertainment (50.0%), money (22.2%), hobby and socializing (19.4%), excitement (12.2%) and distraction (13.9%). Using the SOGS, the results indicated that 72.2% of the sample reported non-problematic gambling (scoring below 3), 11.1% reported problematic gambling (scoring 3–4), and the 16.7% reported probable pathological gambling (scoring 5 or more). The socio-demographic variables of the overall sample are reported in Table 1.

**Table 1:** Socio-demographic variables of the total sample

		**Total sample(*N* = 36)**	
	**Range**	**M (*SD*)**	
**Age**	60–80	67.08 (5.79)	
		***N***	**%**
**Professional status**	Employed	20	55.6
	Unemployed	2	5.6
	Retired	14	38.9
**Education**	Primary School diploma (5 years)	4	11.1
	Middle School diploma (8 years)	6	16.7
	High School diploma (13 years)	19	52.8
	Master’s degree (18 years)	7	19.4
**Marital status**	Single	4	11.1
	Married	27	75.0
	Separated	3	8.3
	Widower	2	5.6

Correlational analysis showed that SOGS score positively correlated with the Uncertainty subscale score of RFQ-8, as well as with all the three subscale scores of the GACS. The SOGS score negatively correlated with Certainty subscale score of the RFQ-8. Significant negative associations were found between the score on the Certainty subscale of the RFQ-8 and scores on both Desire and Relief. The score on the Uncertainty subscale of the RFQ-8 positively correlated with scores for both Desire and Relief (see Table 2).

**Table 2:** Pearson correlation coefficients among measures

	**2**	**3**	**4**	**5**	**6**	**7**	**8**	**9**	**10**
**1. SOGS**	**.581**^**b**^	**.648**^**b**^	**.382**^**a**^	.048	.110	.221	-.157	**-.424**^**b**^	**.609**^**b**^
**2. GACS Desire**	**-**	**.554**^**b**^	**.716**^**b**^	.091	.023	-.059	.050	**-.479**^**b**^	**.368**^**b**^
**3. GACS Anticipation**		**-**	**.378**^**b**^	.001	.046	.019	-.065	-.221	.275
**4. GACS Relief**			**-**	-.045	.061	-.040	.193	**-.512**^**b**^	**.451**^**b**^
**5. Facilitation bias (100 ms)**				-	-.173	-.051	-.088	.130	-.074
**6. Facilitation bias (500 ms)**					-	-.187	-.012	-.306	-.003
**7. Disengagement bias (100 ms)**						-	.013	.027	-.020
**8. Disengagement bias (500 ms)**							-	.088	.000
**9. RFQ-8 Certainty**								**-**	**-.560**^**b**^
**10. RFQ-8 Uncertainty**									-

*SOGS *South Oaks Gambling Screen, *GACS *Gambling Craving Scale, *RFQ-8 *Reflective Functioning Questionnaire

^a^Correlation is significant at the 0.05 level (2-tailed)

^b^Correlation is significant at the 0.01 level (2-tailed)

To identify the potential predictors of older-aged problem gambling, age, scores on both GACS and RFQ-8 subscales, and the facilitation and disengagement bias scores at both 100 ms and 500 ms were entered into a hierarchical regression analysis with problem gambling (SOGS score) as the dependent variable. Because of high VIF value (= 2.8) the GACS Desire subscale was excluded. Results, reported in Table 3, showed that high scores on both GACS Anticipation and RFQ-8 Uncertainty about mental states, and disengagement bias at 100 ms significantly predicted gambling severity among older-age gamblers. The overall model explained 62% of the total variance of the SOGS.

**Table 3:** Summary of hierarchical linear regression analysis with SOGS as the dependent variable

Variable	B	β	*t*	VIF
*Step #1*				
*Age*	.021	.047	.273	1.000
	*R*^*2*^*adj* = *-.027 F(1,34)* = *.074*			
*Step# 2*				
*Age*	.007	.016	.124	1.041
*Anticipation*^*a*^	.980	.587	4.120**	1.167
*Relief*^*a*^	.584	.159	1.115	1.169
	*R*^*2*^*adj* = *.390 F(3,32)* = 8.462**			
*Step #3*				
*Age*	-.029	-.065	-.577	1.041
*Anticipation*^*a*^	.885	.530	4.395**	1.086
*Relief*^*a*^	-.246	-.067	-.458	1.559
*Certainty about mental states*^*b*^	-.287	-.093	-.651	1.663
*Unertainty about mental states*^*b*^	2.182	.454	3.229**	1.613
	*R*^*2*^*adj* = *.570 F(5,30)* = 10.283**			
*Step #4*				
*Age*	-.041	-.092	-.724	1.503
*Anticipation*^*a*^	.815	.489	4.261**	1.224
*Relief*^*a*^	.045	.012	.088	1.758
*Certainty about mental states*^*b*^	.020	.007	.044	2.090
*Unertainty about mental states*^*b*^	2.411	.502	3.698**	1.716
*Facilitation Bias (100)*	.004	.070	.573	1.393
*Facilitation Bias (500)*	.007	.155	1.335	1.253
*Disengagement Bias (100)*	.008	.258	2.430*	1.053
*Disengagement Bias (500)*	-.005	-.147	-1.241	1.311
	*R*^*2*^*adj* = *.624 F(9,26)* = 7.453**			

^a^Gambling Craving Scale

^b^Reflective Functioning Questionnaire

^*^p < .05

^**^p < .01

## Discussion

The aim of the present study was to analyze, for the first time, the role of attentional bias among older-aged male gamblers, specifically focusing on the attentional components and type of biases that can contribute to gambling in old age. Moreover, the present study investigated the role of craving and mentalization, as well as the interplay between the study variables among older-aged gamblers, for the first time. The present study’s results showed that GACS Anticipation, RFQ-8 Uncertainty about mental states, and disengagement bias at 100 ms predicted older-aged problem gambling.

The finding related to the disengagement bias for gambling stimuli when presented at 100 ms indicates that the attentional bias involved in older-aged gambling concerns a difficulty in diverting attention from gambling stimuli in the initial stage of attention, namely the phase in which attention is automatically captured by gambling stimuli, before any processing of the stimulus itself. Therefore, the gambler's attention is held by gambling stimuli so that they took longer to react to the dot, when it appears in the opposite position of the previous gambling cue. This finding clearly indicates that gambling stimuli are valenced to capture gamblers’ attention even when the time of presentation is so short that the individual cannot clearly identify them.

These results corroborate what was previously found in literature among younger adult gamblers [27]. McGrath et al. [15] observed that regular gamblers initially fixated on gambling images, focused on gambling images significantly earlier than neutral ones, and preferentially attended to gambling images for significantly longer periods of time than neutral stimuli. Similarly, Brevers et al. [26] found that individuals with gambling problems showed a difficulty in disengaging attention away from gambling cues, and tended to direct their attention on gambling more frequently than neutral pictures. Kim et al. [55], having observed that problem EGM gamblers preferentially attended to EGM images and had longer fixation times to these images, concluded that individuals with gambling problems could have difficulty shifting attention away from gambling-related stimuli, consistent with incentive sensitization theory [10, 11].

In relation to incentive sensitization theory and the postulated mutual excitatory relationship between attentional bias and craving, despite the predictive role of the anticipation of the pleasure resulting from gambling – Anticipation, a subscale of the GACS – on gambling severity, the present study did not find any association between craving and attentional bias. This was in contrast to some past studies [23, 29, 30], but in line with others [26, 30, 33]. This lack of association could be ascribed to the characteristics of the sample, and, more specifically, to their level of gambling severity.

The studies that have documented the association between attentional bias and craving had a percentage of individuals with gambling problems (50%-67%) much higher than in the present study (28%, which is higher than the prevalence of problem gambling in the general population). Following the suggestion of Young and Wohl [47], because the GACS is able to discriminate different levels of gambling severity, higher craving scores correspond to a higher level of problem gambling. This may reflect the fact that the percentage of individuals with gambling problems in the present study was lower than in the past studies and, consequently, that the SOGS scores varied little between participants [26, 30]. Alternatively, it could be that the relationship between craving and attentional bias changes over time. In the initial phase of gambling involvement, the strong desire to play (craving) is responsible for the gamblers’ attentional polarization towards gambling stimuli, so causing attentional bias. This bias is strategic, because it is driven by the motivational state of craving. This is supported by Ciccarelli et al. [30] who reported a strategic orientation of attention towards gambling among adolescent gamblers, who are likely to have a shorter gambling history than adults.

Over time, and with the repeated exposure to gambling activities, attentional bias becomes automatic, so losing the relationship with the craving. In other words, when the attentional bias becomes automatic, attention may no longer need the motivational drive of craving to be directed towards game stimuli. This explanation is compatible with past studies that (i) have observed an automatic attentional bias among adult gamblers [15, 29, 30], and (ii) did not find an association of craving with attentional bias [26, 30, 33], which is also compatible with the present study’s findings. Indeed, given that more than 80% of the present sample reported having started gambling before the age of 30 years, it is reasonable to assume that a longer gambling history makes strategic bias automatic. This could be the same reason why, in contrast with past studies [25, 28], there was no bias in the maintenance of attention, namely in the phase in which the attention is consciously and strategically oriented towards gambling. In addition, it should be considered that while in the present study both phases of selective attention (initial engagement and maintenance) were investigated, the aforementioned studies have mainly explored attentional maintenance.

With regards to mentalization, the present results corroborate what was previously found concerning the role of compromised reflective functioning as a key risk factor for both adult and adolescent problem gambling [35, 37–39], demonstrating the importance of hypomentalizing in promoting gambling problems from adolescence to old adulthood, across the lifespan. Hypomentalizing refers to the inability to understand the mental states that give rise to behavior, such as desires, wishes, emotions, and intentions. In the specific case of gambling, genuine mentalizing would probably break the loop that leads individuals (often in an impulsive and unconscious manner) to gamble despite negative short- and long-term consequences. The inability to mentalize implies an “opaque” mind, unable to read itself. Under these conditions, it is likely that gamblers feel an uncontrollable urge to gamble (i.e., craving). In support of this, the present study observed significant associations between craving and hypomentalizing, emphasizing that the greater the individual’s inability to understand their own and others' mental states, the greater the levels of craving experienced. It is conceivable that in the presence of compromised mentalization, the individual, not being able to reflect on their own mental states, acts on them. Consequently, it is likely that gamblers with mentalizing deficits act out their craving by gambling.

### Limitations

The present findings should be interpreted in the light of the study limitations. First, the study was correlational, so no conclusion about the cause-effect relationships between the study variables can be drawn. Although an a priori power analysis was performed to estimate the sample size, an effect size of 1 may be too large, and the recruited sample may therefore have been too small. The sample size, as well as the use of convenience sampling, does not allow generalization of the results to the broader population of gamblers. Moreover, the analysis of gender profiles in relation to old age gambling was not possible given that only male participants were recruited. Finally, in relation to the established role of matching gambling cues with the favorite gambling activities (which could have elicited stronger attentional bias among participants; see [15]), another limit relates to not having matched the gambling cues with the gambling activities preferred by participants. This may have resulted in an underestimation of attentional bias scores.

## Conclusions

The present study is the first to assess the role of gambling-related attentional biases, craving, and mentalizing in gambling in older age. The study demonstrated that the profile of older-aged gamblers combines high levels of craving, a difficult to disengage attention away from gambling cues, and an impaired ability to mentalize. Despite the limitations (e.g., all participants being male only), the present findings make an important contribution, in as much as the identification of the variables contributing to problem gambling among older-aged gamblers. The findings will be useful in the development of programs aimed at prevention of gambling addiction in this specific age cohort.

## Data Availability

The data supporting the conclusions of this study are available upon request to the first author, Maria Ciccarelli.

## Footnotes

1 The a-priori power analysis was performed with the program G* Power [43] for the present study. For the regression, having inserted these input parameters: effect size f^2^ = 1.00, α err prob. = 0.05, power = 0.95, and number of predictors = 9, we obtained the following output parameters: critical *F* = 2.30, sample size = 34, actual power = 0.95. The number of participants recruited reflects that required by the power analysis.

2 Although the SOGS is based on the DSM-III criteria, it should be noted that the diagnostic criteria have not changed qualitatively, but quantitatively (the number of criteria, the diagnostic threshold, and the time window for diagnosis has changed). However, the scored items of the SOGS refer to chasing, inability to stop gambling, loss of control over gambling activity, lying about gambling involvement, indebtedness, impaired work, and relationship functioning. In the present study, the time window for the SOGS was the last 12 months, in line with the latest version of the DSM.
